# Self-Powered, Long-Durable, and Highly Selective Oil–Solid Triboelectric Nanogenerator for Energy Harvesting and Intelligent Monitoring

**DOI:** 10.1007/s40820-022-00903-8

**Published:** 2022-08-05

**Authors:** Jun Zhao, Di Wang, Fan Zhang, Jinshan Pan, Per Claesson, Roland Larsson, Yijun Shi

**Affiliations:** 1 Division of Machine Elements, Department of Engineering Sciences and Mathematics, Luleå University of Technology, 971 87 Luleå, Sweden; 2 Department of Engineering and Design, School of Engineering and Informatics, University of Sussex, Brighton, BN1 9RH UK; 3 Division of Surface and Corrosion Science, Department of Chemistry, KTH Royal Institute of Technology, 100 44 Stockholm, Sweden

**Keywords:** Oil, Triboelectric nanogenerator, Energy harvesting, Intelligent monitoring

## Abstract

Triboelectric nanogenerators (TENGs) have potential to achieve energy harvesting and condition monitoring of oils, the “lifeblood” of industry. However, oil absorption on the solid surfaces is a great challenge for oil–solid TENG (O-TENG). Here, oleophobic/superamphiphobic O-TENGs are achieved via engineering of solid surface wetting properties. The designed O-TENG can generate an excellent electricity (with a charge density of 9.1 µC m^−2^ and a power density of 1.23 mW m^−2^), which is an order of magnitude higher than other O-TENGs made from polytetrafluoroethylene and polyimide. It also has a significant durability (30,000 cycles) and can power a digital thermometer for self-powered sensor applications. Further, a superhigh-sensitivity O-TENG monitoring system is successfully developed for real-time detecting particle/water contaminants in oils. The O-TENG can detect particle contaminants at least down to 0.01 wt% and water contaminants down to 100 ppm, which are much better than previous online monitoring methods (particle > 0.1 wt%; water > 1000 ppm). More interesting, the developed O-TENG can also distinguish water from other contaminants, which means the developed O-TENG has a highly water-selective performance. This work provides an ideal strategy for enhancing the output and durability of TENGs for oil–solid contact and opens new intelligent pathways for oil–solid energy harvesting and oil condition monitoring.

**Supplementary Information:**

The online version contains supplementary material available at 10.1007/s40820-022-00903-8.

## Highlights

- The as-designed triboelectric nanogenerator (TENG) generates an excellent electric output, which is an order of magnitude higher than that of TENGs made from commercial dielectric materials.
- The as-designed TENG-based sensor can detect worn debris in oils at least down to 0.01 wt% and water contamination down to 100 ppm, which are much better than other online monitoring methods (particle > 0.1 wt%; water > 1000 ppm).
- A high-selective monitoring system is successfully developed for distinguishing water contamination from the multi-mixed contaminants in lubricating oils.

**Supplementary Information:**

The online version contains supplementary material available at 10.1007/s40820-022-00903-8.

## Graphical abstract

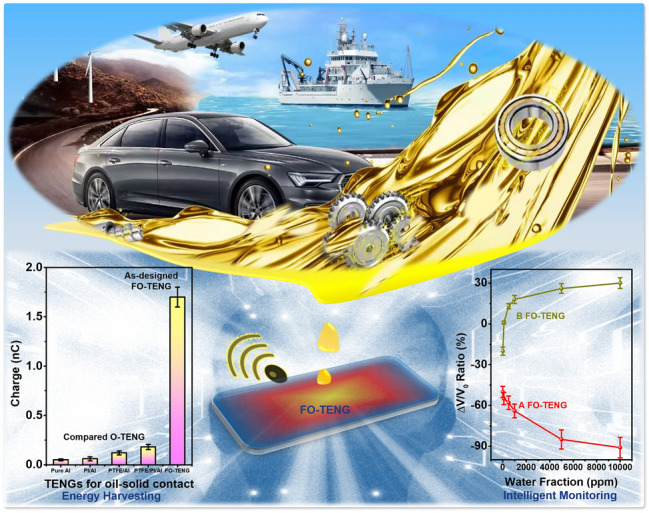

## Introduction

The energy crisis caused by excessive consumption of fossil fuels has become a global problem. Sustainable energy generation is therefore of great interest, and it dramatically influences the development of human civilization and industrialization [1–4]. Traditional energy-harvesting strategies have already been developed based on photoelectric, thermoelectric, piezoelectric, electrostatic, and electromagnetic induction effects. However, such device structures are too complicated for applications, and they are seriously restricted by light intensity, temperature difference [5, 6], etc. On the other hand, various smart machines [7, 8] and electronic devices [9–11] are emerging, which causes an urgent need of self-powered sensors [12, 13] capable to harvest energy from the work environment. The most commercialized portable power sources, batteries, are relatively heavy and face critical lifetime limitations when they are small or flexible [14, 15], and they cannot fulfill the requirement of Big Data and Internet of Things in the coming Industry 4.0 [16]. Triboelectric nanogenerator (TENG), firstly invented in 2012 [17], has attracted worldwide interests due to its simple structure and easy preparation from various commercial materials [18, 19]. It has become a promising technology for energy harvesting and for constructing self-powered sensor systems via utilization of environmental mechanical energy [20–23]. The working mechanism of TENG is based on the coupling of the triboelectrification effect and the electrostatic induction effect, i.e., electrons will flow via the interfaces when two materials contact and subsequently separate [24–28]. Liquid–solid TENGs are expected to have advantage in terms of long service life, which was considered as a severe problem of solid–solid TENGs for practical applications [29–31].

Oils are commonly recognized as the “lifeblood” of industry and are used almost everywhere. For example, using lubricating oils in machinery industry is the most effective way to reduce friction and thus saves energy and reduces resource consumption [32–34]. Proper lubrication will also ensure moving machines working under a safe and healthy condition. The hydraulic oil is a very critical part of any fluid power system and is utilized to transmit force and motion [35]. At the same time, these oils can also provide important information for detecting machine failures, which is similar to the role of human blood indicating health. However, oils often give rise to adsorption and fouling problems, which will cause a lot of issues in different applications and it may even be detrimental for human life. For example, face masks for capturing PM2.5 or preventing COVID-19 have low efficiency in the surroundings containing oil vapor due to adsorption [36]. Smart wearable electronics lay a good foundation for the rapid development of an intelligent world. However, the damage caused by oil stains from human–electronic interfaces has always been a thorny issue [37], which not only affects the aesthetics of materials but also corrodes and affects the performance of electronic circuits.

In our recent work [38], we prepared an O-TENG based on the contact and separation between lubricating oils and solid surfaces to obtain triboelectric signals, which can be used for online monitoring lubricating oils conditions. It is found that oils are prone to strongly adsorbing on the contact solid surface, resulting in lower signal output. This irreversible oil-interfacial adsorption issue will, in the long run, reduce the service life of the O-TENG. In comparison with water–solid TENG, O-TENG generates significantly lower signal output [39]. This makes it much more challenging to use O-TENGs as an energy-harvesting technology or as self-powered sensors. We also noticed that it is very difficult to distinguish different contaminants in oils based on the previous O-TENG made from commercial materials. Identifying contaminant types is actually a great challenge for other types of oil monitoring sensors [40–43].

In this study, a self-powered, long-durable, and highly selective O-TENG for oil–solid contact is prepared by developing and using a smart coating with controlled surface wetting properties. The prepared coating has higher electron-withdrawing ability and better oil adsorption resistance compared to O-TENGs constructed from commercial materials, e.g., polytetrafluoroethylene (PTFE) and polyimide (PI). The developed O-TENG displays high-output and long-durable performance. In addition, the feature of the controlled surface wetting properties not only gives the developed TENG the capability of monitoring the contaminants in oils precisely, but also gives the possibility to specifically identify water contamination. This study will open new intelligent pathways for both oil–solid energy harvesting and accurate oil quality monitoring.

## Experimental Section

### Materials

Anionic fluorocarbon surfactant (Capstone FS-61, noted as Fc) was provided by DuPont. 1H, 1H, 2H, 2H-Perfluorodecyltriethoxysilane (noted as Fs), hydrophilic nano-SiO_2_ (with an average particle size of 40 nm), and paraffin oil were supplied by Aladdin. Engine lubricant oil (0W-16) was obtained from MOTUL. Deionized (DI) water and other chemical reagents were purchased from Sinopharm Chemical Reagent Co., Ltd. All the materials and chemicals were used as-received without any treatment.

### Preparation of PI/Al, PTFE/Al, PTFE/PI/Al O-TENGs

A commercial 3 M double tape (3 × 3 cm^2^) is used as the substrate of O-TENG in this work. A PI single tape was directly placed on the 3 M double tape to avoid leakage of electricity, and an Al electrode tape with 2 cm width was pasted on the PI surface. For the O-TENGs based on commercial dielectric materials, each surface of the Al electrode was completely covered with an 80-μm-thick PI film (PI/Al O-TENG) or PTFE film (PTFE/Al O-TENG). The edges of the electrode were sealed by the film. In addition, another O-TENG based on the mixed dielectric materials (PI and PTFE) was prepared, whose Al electrode surface was completely covered with a PI tape with glue on two sides firstly and then covered by the PTFE film (PTFE/PI/Al O-TENG).

### Fabrication of FO-TENG

SiO_2_ nanoparticle was firstly dissolved in deionized water. The ratio of SiO_2_: water is 1: 30 g. After 10 min stirring, Fc was dissolved in the SiO_2_ solution that was kept under stirring for 1 h to fabricate the waterborne solution. The fraction of materials for preparing the Fc-modified nano-SiO_2_ suspension is shown in Table S1. The prepared SiO_2_ suspension was sprayed on the film surfaces and dried in air, and the spray process was repeated 5 times to get the final Fc-SiO_2_ coating. The coating was then put into a chamber (12.5 L), and Fs was allowed to deposit from air at 80 °C for 30 min. The fraction of Fs is shown in Table S2.

### Characterization

The morphologies of all O-TENGs were measured by using a three-dimensional (3D) white-light interferometry microscopy (Nexview, ZYGO Lambda, USA) and a scanning electron microscopy (SEM, Quanta 200 FEG, FEI, USA) under secondary electron conditions with an accelerating voltage of 5 kV. The chemical composition of all O-TENGs was investigated by Fourier transform infrared spectroscopy (FTIR, Vertex 70 V, NETZSCH, Germany) and X-ray photoelectron spectroscopy (XPS, PHI Quantera II, Ulvac-Phi Inc., Japan) operating with Al Kα radiation under a vacuum pressure of 10^−7^ Pa. The increased Young’s modulus of O-TENGs was examined by an atomic force microscopy (AFM, ICON, Bruker, Germany) and analyzed using the Hertzian (spherical) mode. Contact angle was determined at 20 ℃ using an Attension Theta optical tensiometer (Biolin Scientific, Sweden) and 5 μL droplets of distilled water or paraffin oil. All O-TENGs signals were measured by an electrometer Keithley 6514. The oil tank was driven by a linear motor (LinMot, Switzerland).

## Results and Discussion

### Physical–chemical Structure of the Developed O-TENG

**Fig. 1:**
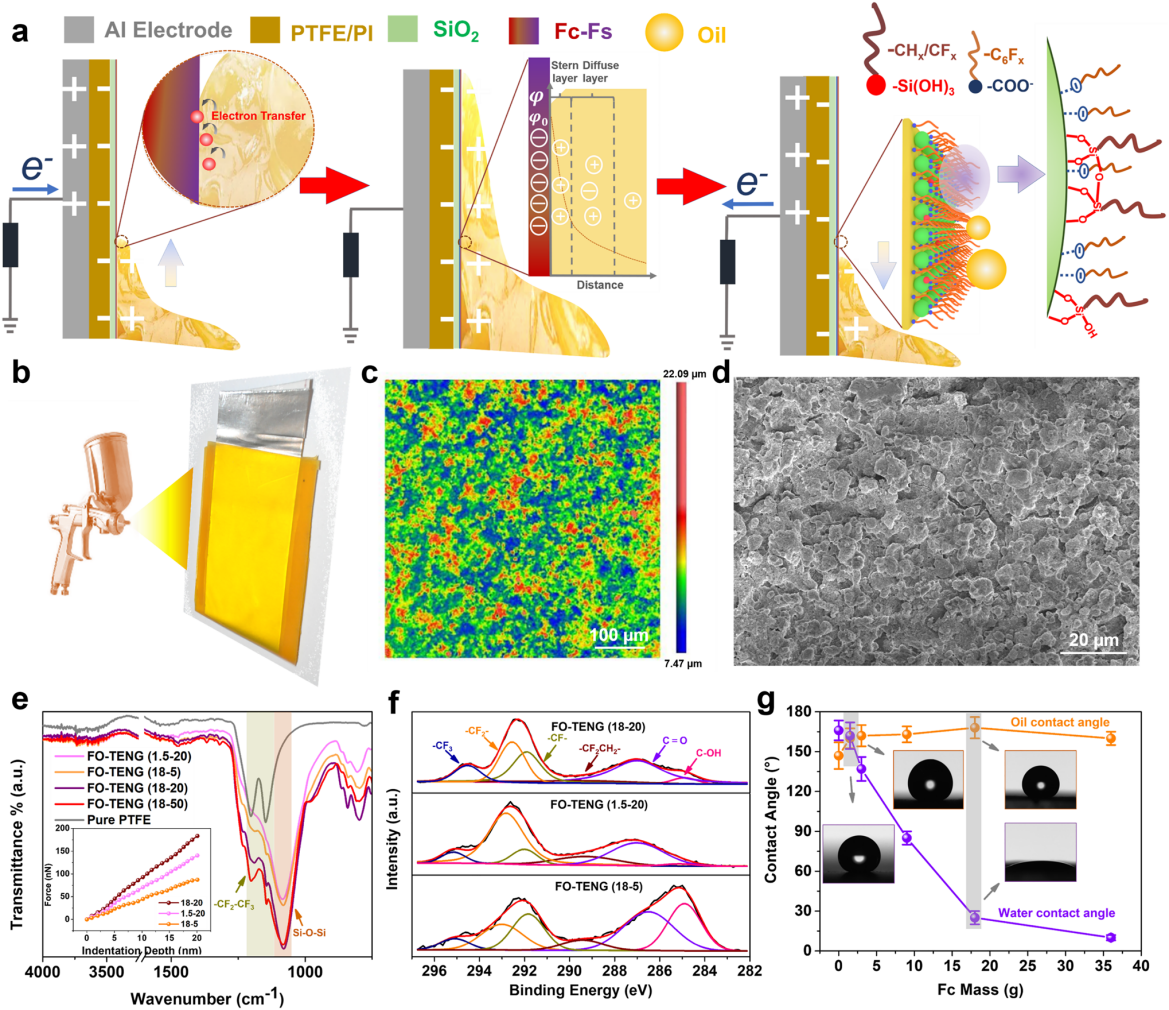
Physical–chemical structure of the developed O-TENG. **a** Schematic illustration on the triboelectric mechanism of the FO-TENG. **b** Illustration schematic of the spray process. **c, d** Two-dimensional and SEM morphologies of the FO-TENG surface (18–20). **e** FTIR spectra of the FO-TENG, inset: the force–displacement curves as pressed by a probe. **f** C 1*s* XPS spectra of the FO-TENG surface. **g** Contact angles (oil and water) of FO-TENGs as a function of Fc mass (Fs: 20 mg), inset: paraffin oil and deionized water contact angle images

The structure of the prepared O-TENG is shown in Fig. 1a and Fig. 1b. The O-TENG modified with different amounts of Fc(x) and Fs(y) is noted as FO-TENG(x–y). For example, one modified by 18 g Fc and then deposited in 20 mg Fs air is noted as FO-TENG (18–20). The O-TENGs based on a commercial dielectric material (PI or/and PTFE) and an Al electrode are also prepared for comparison (noted as Pure Al, PI/Al, PTFE/Al and PTFE/PI/Al). As shown in Fig. 1c-d, the FO-TENG displays a hierarchical cellular-like structure and has a surface roughness of ~ 4 µm.

To verify the chemical structure of the FO-TENG, FTIR, and XPS spectra were employed (Fig. 1e-f). The FO-TENG shows typical absorption peaks at 1084 cm^−1^ (Si–O-Si stretching) and 1140–1200 cm^−1^ (C-F stretching) [44] in FTIR spectra. When higher amount of Fs (20 or 50 mg) is used, the C-F stretching peaks become more obvious and the FO-TENGs show enhanced intensity of Si–O-Si absorption peak. Mechanically, the FO-TENGs prepared from larger fraction of Fs (20 mg) have higher elasticity modulus (113 and 100 MPa) than the one with smaller fraction of Fs (5 mg) (60 MPa) (inset of Fig. 1e). Based on the above information, we can confirm that Fs is successfully deposited on the surface via a strong covalent bond, which improves mechanical properties of the coating. Figure 1f shows the high-resolution XPS spectra of the surface of FO-TENG and the C 1*s* signal can be deconvoluted into six peaks at 285.4, 286.9, 289.4, 291.7, 293, and 295 eV, assigned to C–O/C–OH, C = O, –CF_2_CH_2_–, –CF_2_–, and –CF_3_, respectively [44, 45]. The intensity of the C–O/C–OH peak decreases, while the intensities arising from the fluorocarbon chains (–CF_2_CH_2_–, –CF_2_–, and –CF_3_) increase when a higher fraction of Fs is used. In general, according to the XPS wide-scan spectra, Fc makes a bigger contribution to the F content at the surface of the coating, and the F content in all the FO-TENG increases slightly after deposited in Fs air (Fig. S1 and Table S3). It can also be seen that the F surface content of FO-TENG (18–20) is significantly high, which will show a strong electron-withdrawing ability.

It can be seen from Figs. 1g and S2 that the wetting behavior of FO-TENG is greatly influenced by Fc. The FO-TENG achieves superoleophobic properties when the added Fc is more than 1 g, e.g., the FO-TENG (18–20) has an oil contact angle (CA) of 167°. In contrast, the PI and PTFE surfaces are oleophilic with oil contact angles of 53° and 58°, respectively (Fig. S3). Further, the water CAs of both PI and PTFE are lower than 120°. The presence of hydrophilic units (–COO– groups) from Fc (Fig. 1f) and migration of Fc to the air–water interface of the droplet, the water CA of FO-TENG decrease gradually with increasing Fc fraction (Fig. 1g), while the water CA increases with the increase of the Fs fraction (Fig. S2). This is because the deposited Fs induces the oleophobic chains of Fc (−C_6_F_x_) to rearrange towards the top surface [46], which blocks the subsurface hydrophilic units of Fc and explains why the FO-TENG (1.5–20) has a stable superamphiphobic surface property (Fig. 1g).

### Electrical Output Performance of O-TENG

**Fig. 2:**
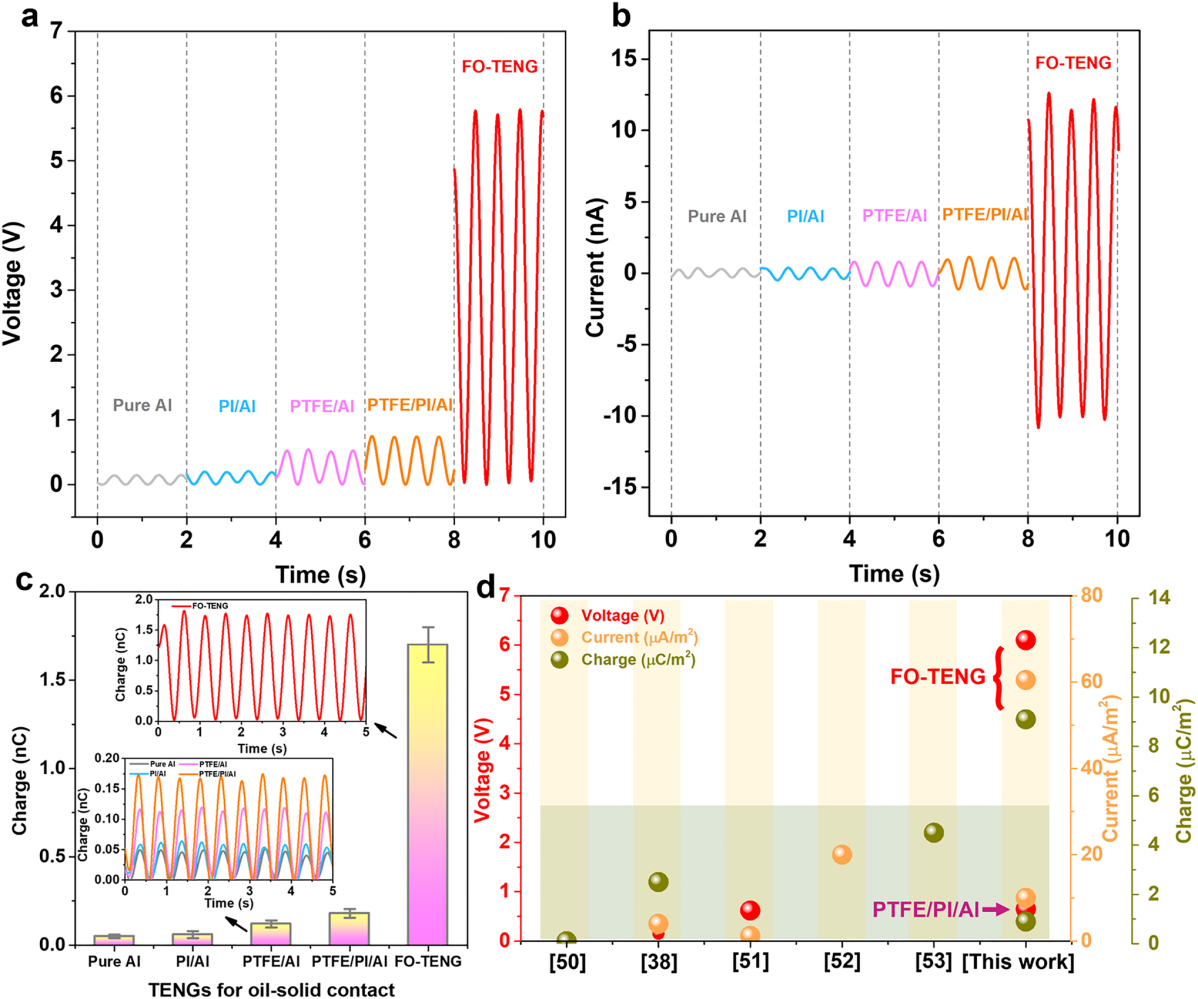
Electrical output performance of O-TENG. **a-c** The open-circuit voltage, short-circuit and transferred charge curves of the FO-TENG (18–20) compared with other O-TENGs for oil-solid contact. **d** Comparison of the triboelectric output performance for oil–solid contact in past years

The O-TENGs (3 × 3 cm^2^) are attached to an inner wall of an oil tank to convert the oil wave energy to electrical power (Video S1). The triboelectric charges are generated by the periodical oscillation of the oil in contact with and separated from the O-TENG surfaces recorded via a single electrode mode (Fig. 1a). The as-designed FO-TENG has a significantly enhanced triboelectric performance and generates an excellent electric output (wave frequency ~ 2 Hz) with an open-circuit voltage of 6.0 V, a short-circuit current of 12 nA, and a transferred charge of 1.82 nC (charge density of 9.1 µC m^−2^) as shown in Fig. 2a–c. It can also be seen from Fig. 2a–b that the voltage and current peaks of the Pure Al, PI/Al, and PTFE/Al TENGs are lower than 0.5 V and 2 nA, respectively. The PTFE/PI/Al O-TENG shows a little higher output of voltage and current at about 0.65 V and 0.25 nA due to intermediate layer effect [47]. The transferred charges of all these O-TENGs (Pure Al, PI/Al, PTFE/Al and PTFE/PI/Al) are less than 0.17 nC (charge density of 0.85 µC m^−2^). The triboelectric performance of the as-designed FO-TENGs is thus an order of magnitude higher than other O-TENGs made from commercial PTFE and PI materials. Normally, PTFE is considered as the commercial material that has the strongest ability of gaining negative triboelectric charge, which in principle should generate high output signals. Our surface modification strategy for FO-TENGs can also be applied on different commercial materials, and all of them enable high output (Fig. S4).

Electron transfer is the dominating mechanism for triboelectrification process in solid–solid cases, so the paraffin oil with no ions can generate a certain amount of charges by electron transfer between oil–solid interfaces [39]. Since a solid material with high electron-withdrawing capability is used here, the electron is transferred from oil to the surface when oil contacts the solid surface (Fig. 1a), making its surface to be negatively charged. After that, an electrical double layer (EDL) is formed in order to neutralize the charged solid surface and, in turn, results in a potential between the surface and the oil. Normally, the EDL consists of a Stern layer and a diffuse layer, which are occupied by immobile counterions and layer, respectively [48]. Thus, the enhanced electric output partly originates from the electron-withdrawing ability of the functional groups on the solid surface, which is due to the high content of F atoms on the surface providing a strong electron-withdrawing ability (Table S3). It is found that oils with low surface energy can be easily adsorbed on PTFE, glass and polyethylene surfaces, which will cause electric-field screening effect due to residual oils and resulting in a quick decrease of the output signal [38]. Fortunately, the as-designed FO-TENG has a superoleophobic surface, which ensures a much weaker electric screening effect and thus achieving a higher signal output.

The voltage output magnitude of the FO-TENG increases with the increasing reciprocating frequency of the wave in the oil tank (Fig. S5a). The reason is that the wave height is linearly related to reciprocating frequency (Fig. S5b), resulting in a bigger oil–solid contact area that enhances the output signal. The average voltage output of the FO-TENG increases from 0.7 to 6.0 V by increasing the wave height from 0.1 (0.25 Hz) to 1 (2 Hz) cm. According to the theory of a signal-electrode mode TENG [49], the open-circuit voltage is related to the amount of transferred charges Q, i.e., Q = σ_0_S = σ_0_ωx, V(x) = k_q_Q_(x)_ = k_q_ωx. Here, V(x) is the open-circuit voltage; k_q_ is a correlation factor; ω is the width of the electrode; x is the instantaneous oil wave height; and σ_0_ relates to the surface charge density. It means that the output voltage of the FO-TENG is expected to vary linearly with the instantaneous wave height, which is in good agreements with the result from Fig. S5c. To get a fair comparison, the following results are based on a wave height of 1 cm (the contact area ~ 2 cm^2^). The parameter σ_0_ mainly relates to the surface charge density, which can be enhanced by Fc and Fs. It is seen that with the increase of the Fc fraction, the signal output increases significantly (Fig. S6). The output also increases by using higher amounts of Fs until it reaches 20 mg, where the output reaches a high plateau value. The optimum masses of Fc and Fs are therefore, respectively, 18 g and 20 mg (Fig. S6). In addition, with the increase of temperature, the output voltage is slightly decreased because the high temperature can cause thermionic emission, which is in good agreement with our recent study [38], but the output performance is relatively stable when the temperature ranges from 25 to 50 °C. The performance of representative oil–solid triboelectric output was shown in previous works (Fig. 2d) [38, 50–53]. It can be seen that the FO-TENG developed in this work exhibits an obvious superior voltage, current density, and charge density.

### Durability and Energy Harvesting of O-TENG

**Fig. 3:**
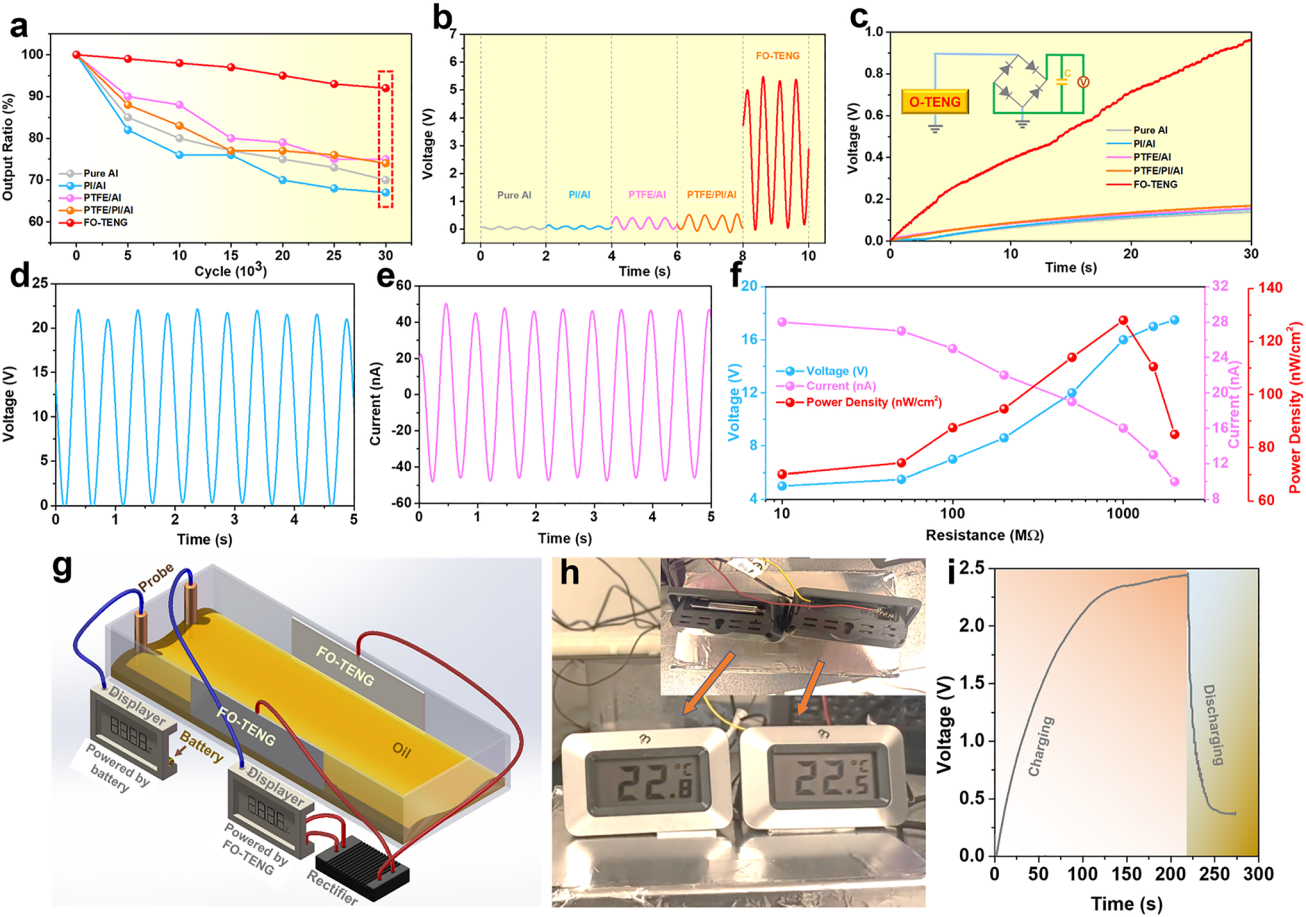
Durability and energy harvesting of O-TENG. **a** The long-term durability of the FO-TENG (18–20) for oil-solid contact. **b** Comparison of voltage output of TENGs. **c** Voltage accumulated across the capacitors with the capacitances of 4.7 μF, inset: equivalent circuit model of the bridge rectifier to convert the alternating signals. **d, e** The open-circuit voltage and the short-circuit of the large-sized FO-TENG (Fig. S12) based on double-electrode mode. **f** The peak current, voltage and power of the large-sized O-TENG with various load resistances. **g** Two commercial monitor displays for real-time and online monitoring of the oil temperature are, respectively, powered by a battery and the large-sized FO-TENG. **h** Real image of the monitor displays showing the online temperature of oil. **i** Charging/discharging curve of the large-sized FO-TENG for powering the commercial display

The as-designed FO-TENG not only generates a high output, but also has a much better durability than other O-TENGs (Fig. 3). The FO-TENG maintains a very high output ratio of the real-time voltage (*V*_t_) to the original value (*V*_o_) under continuous operating cycles (Fig. 3a). The output ratio (*V*_t_/*V*_o_) is more than 90% even after 30,000 cycles, and the voltage value of the O-TENG is as high as 5.5 V, which are much higher than those of Pure Al, PI/Al, PTFE/Al and PTFE/PI/Al TENGs (lower than 0.5 V) (Fig. 3b) at the operation frequency of 2 Hz. The O-TENGs based on commercial materials, i.e., Pure Al, PI/Al, PTFE/Al, and PTFE/PI/Al, have low oil CAs (lower than 50°, Fig. S3), which leaves residual oil adsorbed on the surfaces during the oil–solid contact and separation process (Fig. S7), resulting in decreasing signal output and lower durability [38]. There is almost no oil residue left on the surface of FO-TENG (Fig. S7). Due to no direct solid–solid friction/contact, the hierarchical cellular-like structure of the FO-TENG is almost intact after long-term working cycles (Fig. S9) and the FO-TENG (18–20) has a superoleophobic property after 30,000 cycles (Fig. S10). It means the FO-TENG has a high durability. The oil contact angle of the FO-TENG decreases with the increase of working cycles probably because of some Fc/Fs molecular degraded or dropped from TENG surface (Fig. S11). Considering the advantages on durability and signal output, the FO-TENG was used for energy harvesting in the following part.

In order to study the energy-harvesting capability, the FO-TENG was utilized to charge commercial capacitors by using a full-wave rectifier. It can be seen in Fig. 3c that the capacitor is charged to 1 V within 30 s by the FO-TENG, while the other O-TENGs only charge the capacitor up to about 0.1 V. As known, the contact area and contact mode play an important role in the output performance of TENGs. For improving the FO-TENG triboelectric capability, we studied a large FO-TENG (contact area ~ 8 × 1 cm^2^) for energy harvesting based on the same single-electrode mode (Fig. S12). The output performances of this FO-TENG (Fig. S13) are much higher than the FO-TENG with a relatively small electrode (Fig. 2) (contact area ~ 2 × 1 cm^2^). Accordingly, a double-electrode mode of FO-TENG is further proposed (Fig. 3d–e), and the open-circuit voltage and the short-circuit voltage of this FO-TENG are, respectively, as high as 22.5 V and 50 nA due to its larger oil–solid contact area and its double-electrode working mode. The peak voltage, current density, and power density of this large FO-TENG with various load resistances are shown in Fig. 3f. As the load resistance increases, the voltage is raised, whereas the current is reduced. At a load resistance of 1000 MΩ, the maximum output power density (1.23 mW m^−2^) is achieved. The as-designed large-size FO-TENG was also employed to harvest oil wave energy for powering a thermometer display (Fig. 3g and Video S2). For comparison, another displayer powered by a commercial battery is also included (Fig. 3h). As shown in Fig. 3i, the FO-TENG can charge the capacitor (33 µF) to a voltage of ~ 2.5 V within 220 s, which can power the thermometer for around 10 s, which is high enough for many different self-powered sensor applications. The above work proves that the FO-TENG has high output performance and long-term durability which can be employed as a power source in oily environment.

### On-line and Smart Monitoring Based on O-TENG

It is commonly believed that high-sensitive, on-line monitoring of lubricating oils is essential for the development of smart machines. As we described above, the O-TENG sensor for on-line lubricant oils condition monitoring developed in our recent work [38] has the problems of low signal output, short service life and difficulty of distinguishing the contaminant types. Interestingly, the as-designed FO-TENG has the potential to solve these challenges, as will be shown next.

**Fig. 4:**
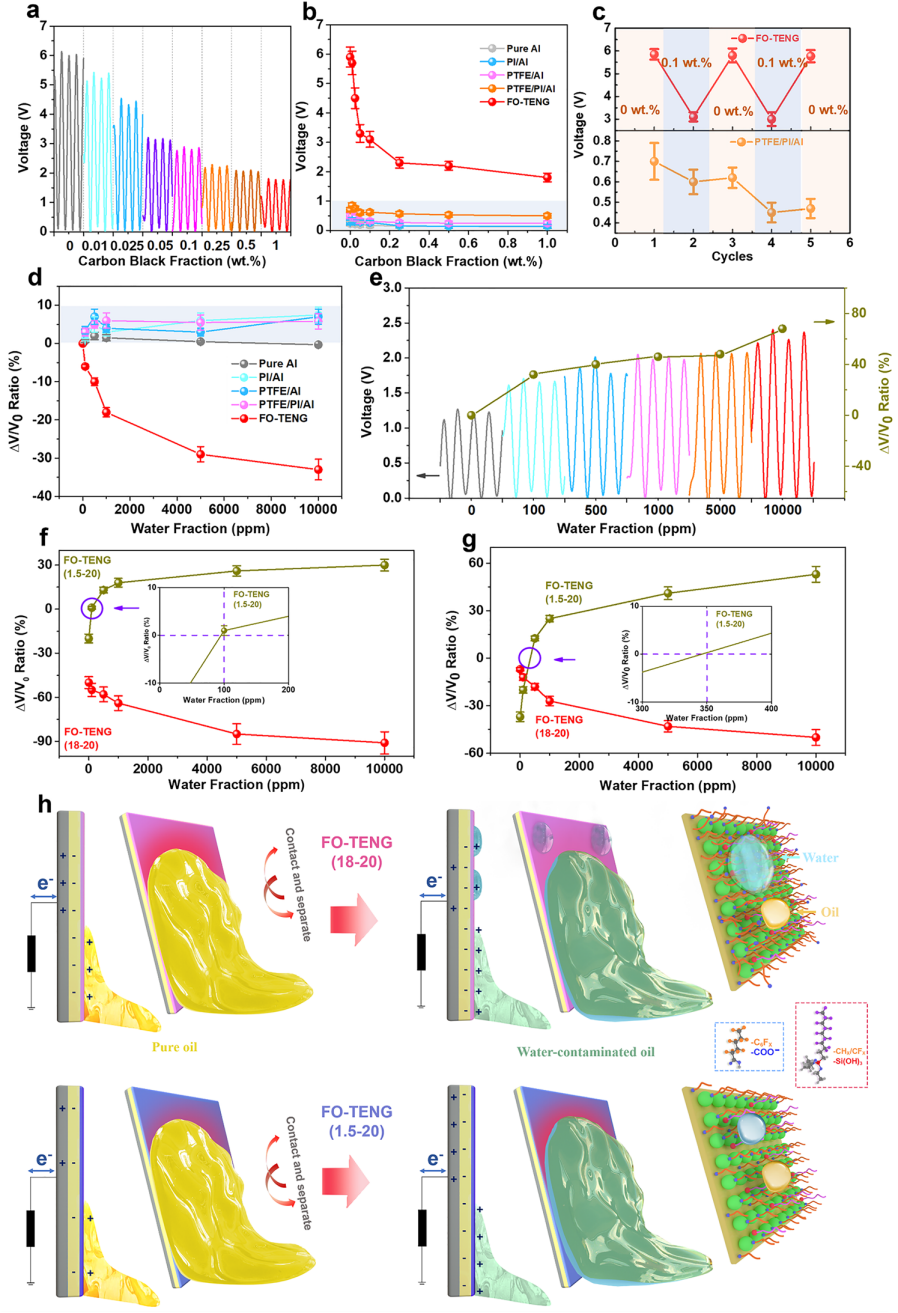
Online and smart monitoring based on O-TENG. **a, b** The voltage output of the carbon black-ladened paraffin oil. **c** Reversible self-cleanability of the FO-TENG (18–20) compared with the O-TENG (PTFE/PI/Al). **d** Comparison of normalized voltage output of O-TENGs contacting the water-ladened paraffin oil. **e** The voltage output of the FO-TENG (1.5–20) contacting the water-ladened oil. **f** The voltage outputs of FO-TENGs identifying the water fraction in a contaminant-ladened paraffin oil (carbon black: 0.1 wt%). **g** The voltage outputs of FO-TENGs identifying the water fraction in a commercial contaminant-ladened lubricating oil. **h** Schematic of the FO-TENG systems for identifying intruded water in oils by surface wetting property design

It can be seen from Figs. 4a and S14 that with increasing particle contamination, the voltage output decreases dramatically. In particular, a clearly changed voltage value is found already at a low particle concentration (0.01 wt%). This means that the FO-TENG has a very high sensitivity (from carbon deposition and worn debris), which is much better than previous works for monitoring of the particle contaminants in oil (> 0.1 wt%) [38, 41, 42]. Thus, the as-designed FO-TENG is able to achieve high-sensitive, real-time and online monitoring of the quality of lubricating oils. On the contrary, the O-TENGs based on commercial dielectric materials (PI and PTFE) are not able to accurately monitor the particle contaminates in the oil. It is believed that the problem with these O-TENGs is due to the residual oils/contaminates on the surface resulting in the electric-field screening effect. The FO-TENG has good self-cleanability in an oily environment because of its oleophobic surface characteristics (Video S3). To further verify the self-cleaning property of the as-designed FO-TENG, the FO-TENG was exposed to different oil environments (pure oil and contaminated oil) as shown in Fig. 4c. It is found that the output signal decreases significantly as the FO-TENG was exposed to the contaminated oil (carbon black 0.1 wt%), but the voltage immediately increases after being exposed to a pure oil environment. After several cycles, the voltage output of the FO-TENG is back to normal (as high as 5.85 V). The self-cleanability of the FO-TENG is also confirmed according to the results of Raman spectra (Fig. S15). In contrast, the voltage output of PTFE/PI/Al O-TENG always decreases with time after being in contact with pure or contaminated oil (Fig. 4c).

Water/moisture contaminations easily permeate oils by coolant leakage or water condensation from the ambient environment [54–56]. This provides a high risk to damage the oil quality and to corrode the machine. Only 1 wt% water in oil can shorten the rated service life of a machine element by almost 90% [56]. It is difficult to identify and determine the exact content of water in the contaminated oil [38], which in practice, simultaneously, contains intrusive water, deposited carbon and wear debris. Until now, there is not a proper monitoring system or sensor with a high sensitivity to distinguish water from other contaminates, which thus remains a great challenge. Most available sensors can only monitor one specific contaminant with a relative low sensitivity, which is inadequate to assess lubricants performance for predicting the health status of a machine [41, 43, 56]. It can be seen from Fig. 4d that the output signal of O-TENGs (Pure Al, PI/Al, PTFE/Al and PTFE/PI/Al) is increased only by around 10% when the water concentration increases from 0 to 10,000 ppm (1 wt%). The sensitivity of these O-TENGs is too low to monitor the intrusive water in oils. For our as-designed O-TENG, the FO-TENG (18–20) has a good hydrophilicity (Figs. 1g and S2). The voltage output of the FO-TENG (18–20) is strongly reduced when the TENG in contact with the 100 ppm water-contaminated oil as shown in Fig. 4d, which is caused by water adsorption and wetting on the FO-TENG (18–20) (Fig. 1g). In addition, the FO-TENG (18–20) can be used repeatedly for water-contaminated oil. The output signal decreases as the FO-TENG is exposed to the water-contaminated oil (1000 ppm), but the voltage immediately increases after being exposed to a pure oil environment (Fig. S16). Although there is a certain hydrophilicity Fc groups (-COOH) in the FO-TENG, the fraction of water in oils is low and the physical interaction between water and the surface is weak. The microstructure of the FO-TENG cannot be influenced by the moisture, i.e., water contaminant when the water fraction is relatively low (~ 1000 ppm) (Fig. S17). When the water contaminant in oil is much higher (~ 10,000 ppm), the FO-TENG also can repeat the triboelectric performance but the output value is decreased because of the much adsorption of water on the contact surface because some cracks of the hydrophilic SiO_2_ coating occur after three cycles, which is resulted from the surface wetting and accumulating of water (Fig. S17). Water levels of 500 ppm are known to be concerning, and concentrations of 1000 ppm are thought to be critical [57–59]. It means high fraction of water in oils (> 1000 ppm) is not allowed and could not happen in practical machine systems. Thus, the self-cleaning capability for water contaminated ranging from 0 to 1000 ppm in oils is enough and meaningful for many practical applications.

In addition, the FO-TENG (1.5–20) displays superhydrophobicity (Fig. 1g). The voltage output of the FO-TENG (1.5–20) instead increases with increasing water content in the oils (Video S4). For instance, the voltage signal output of the FO-TENG (1.5–20) is increased by 27% when 100 ppm water is added in the oils. The reason is that the triboelectric effect of oil is enhanced (better electron withdraw capability) by the added water (Fig. 4e). Therefore, the FO-TENG can detect water contamination at least down to 100 ppm for real-time and online monitoring, which is much better than other online water-monitoring methods (> 1000 ppm) [40, 43, 56]. Accordingly, to identify water contaminant from other contaminants, we propose a new novel route to monitor and identify water from concurrent-intruded contaminants based on the two types of FO-TENGs. As shown in Fig. 4f, in contact with the concurrent contaminant-laden oils, the output signal of the FO-TENG (18–20) decreases with increasing amount of added water (Fig. 4h), while that of the FO-TENG (1.5–20) instead increases. It is worth noting that the intruded water can be successfully identified and monitored when the output ratio (∆V/V_0_) of the FO-TENG (1.5–20) displays a positive value (> 0). It means that the selective sensitivity of water in a contaminant-laden paraffin oil is about 100 ppm (inset of Fig. 4f).

Finally, as a demonstrative work, a fresh commercial fully formulated lubricating oil with various kinds of water and carbon black is used as a test object. In contact with the particle contaminant-laden oils, the output signal of the FO-TENG decreases (Video S5). As expected, the voltage ratio of the FO-TENG (18–20) decreases significantly with increasing water fraction (Figs. 4g and S18a), and the added water generates a higher triboelectric ability for the FO-TENG (1.5–20) (Figs. 4g and S18b, Video S6). The ratio value of the FO-TENG (1.5–20) increases from − 37% to 12.5% when 500 ppm water is intruded to the contaminated lubricating oil. The output voltage of the FO-TENG (1.5–20) is increased by as much as 100% at a high fraction of water (10,000 ppm) (Fig. S18b). It is also found that the developed monitoring method has a selective sensitivity of water at 350 ppm (inset of Fig. 4g) in a commercial contaminated oil (carbon black 0.1 wt%). As shown in Fig. 4h, the FO-TENG (18–20) can generate a much higher electrical output in the oil–solid interface because of the low surface energy groups of Fc and Fs. The hydrophilic groups (–COO–) from Fc provide an effective capturing of water molecular. Once the FO-TENG surface contacts water, the output signal will decrease due to the screen effect from adsorbed water on the contact surface. For the FO-TENG (1.5–20) with a very low Fc, the deposited Fs easily induces the oleophobic chains of Fc (–C_6_F_x_) to rearrange toward the top surface, resulting in a superamphiphobic surface property (Fig. 1g). Thus, the voltage output of the FO-TENG (1.5–20) instead increases when contacting water-contaminated oils. Therefore, it is proved that the as-designed water-selective monitoring system (FO-TENGs) can be used for intelligent diagnosing of oil conditions. This work provides an ideal strategy for enhancing the electrical output and durability of TENGs for oil–solid contact and opens new intelligent pathways for both energy harvesting from oil environment and intelligent oils condition monitoring.

## Conclusion

In summary, energy harvesting and intelligent oils condition monitoring have been achieved based on FO-TENGs through engineering of solid surface wetting properties. FO-TENGs display high-output and long-durable performance because of their enhanced electron capture capability and strong oil adsorption resistance compared with O-TENGs based on commercial PI and PTFE materials. The as-designed FO-TENG generates an excellent electric output with an open-circuit voltage of 22.5 V, a short-circuit current of 50 nA, a charge density of 9.1 µC m^−2^ and a power density of 1.23 mW m^−2^. The achieved electricity output is an order of magnitude higher than that of O-TENGs made from commercial dielectric materials. The FO-TENG displays excellent durability and maintains 90% of the initial electrical output even after 30,000 cycles, which is much higher than achieved by O-TENGs based on PTFE or PI (70%). A FO-TENG with a size of 8 × 2 cm^2^ can charge a capacitor (33 µF) up to a voltage of ~ 2.5 V within 220 s, which successfully powers a digital temperature display from the energy harvested from oil waves. The FO-TENG can be used for real time and online monitoring of the quality of lubricating oils. It has very high sensitivity as it can detect debris at least down to 0.01 wt% and water contamination down to 100 ppm, which are much better than other previous online monitoring methods (particle > 0.1 wt%; water > 1000 ppm). Furthermore, a high-selective monitoring system based on two types of FO-TENGs for identifying water contamination is successfully developed. The intruded water can be successfully distinguished from the multi-mixed contaminants. Thus, the FO-TENGs designed in this work are of great value for energy harvesting from oil movements and intelligent monitoring of lubricating oils.

## Supplementary Information

Below is the link to the electronic supplementary material.

Supplementary file1 (MP4 6633 KB)

Supplementary file2 (MP4 9285 KB)

Supplementary file3 (MP4 3576 KB)

Supplementary file4 (MP4 10737 KB)

Supplementary file5 (MP4 6679 KB)

Supplementary file6 (MP4 11983 KB)

Supplementary file7 (PDF 1858 KB)

## References

[1] Xiao CF, Kim JH, Cho SH, Park YC, Kim MJ (2021). Ensemble design of electrode-electrolyte interfaces: toward high-performance thin-film all-solid-state li-metal batteries. ACS Nano.

[2] Fu S, He W, Tang Q, Wang Z, Liu W (2022). An ultrarobust and high-performance rotational hydrodynamic triboelectric nanogenerator enabled by automatic mode switching and charge excitation. Adv. Mater..

[3] Askari H, Khajepour A, Khamesee MB, Saadatnia Z, Wang ZL (2018). Piezoelectric and triboelectric nanogenerators: trends and impacts. Nano Today.

[4] Slabov V, Kopyl S, Santos MPS, Kholkin AL (2020). Natural and eco-friendly materials for triboelectric energy harvesting. Nano-Micro Lett..

[5] Zhu J, Zhu M, Shi Q, Wen F, Liu L (2020). Progress in teng technology-a journey from energy harvesting to nanoenergy and nanosystem. EcoMat.

[6] Yang B, Lee C (2009). A wideband electromagnetic energy harvester for random vibration sources. Adv. Mater. Res..

[7] Tabor DP, Roch LM, Saikin SK, Kreisbeck C, Sheberla D (2018). Accelerating the discovery of materials for clean energy in the era of smart automation. Nat. Rev. Mater..

[8] Hu Y, Xu C, Zhang Y, Lin L, Snyder RL (2011). A nanogenerator for energy harvesting from a rotating tire and its application as a self-powered pressure/speed sensor. Adv. Mater..

[9] Kwon S, Kim H, Choi S, Jeong EG, Kim D (2018). Weavable and highly efficient organic light-emitting fibers for wearable electronics: a scalable, low-temperature process. Nano Lett..

[10] Xi Y, Hua J, Shi Y (2020). Noncontact triboelectric nanogenerator for human motion monitoring and energy harvesting. Nano Energy.

[11] Yang Y, Huang Q, Niu L, Wang D, Yan C (2017). Waterproof, ultrahigh areal-capacitance, wearable supercapacitor fabrics. Adv. Mater..

[12] Wu J, Xi Y, Shi Y (2020). Toward wear-resistive, highly durable and high performance triboelectric nanogenerator through interface liquid lubrication. Nano Energy.

[13] Zhu M, Yi Z, Yang B, Lee C (2021). Making use of nanoenergy from human - nanogenerator and self-powered sensor enabled sustainable wireless iot sensory systems. Nano Today.

[14] Larcher D, Tarascon JM (2015). Towards greener and more sustainable batteries for electrical energy storage. Nat. Chem..

[15] Seol ML, Jeon SB, Han JW, Choi YK (2017). Ferrofluid-based triboelectric-electromagnetic hybrid generator for sensitive and sustainable vibration energy harvesting. Nano Energy.

[16] Wen F, Zhang Z, He T, Lee C (2021). AI enabled sign language recognition and VR space bidirectional communication using triboelectric smart glove. Nat. Commun..

[17] Fan FR, Tian ZQ, Wang ZL (2012). Flexible triboelectric generator. Nano Energy.

[18] He W, Liu W, Chen J, Wang Z, Liu Y (2020). Boosting output performance of sliding mode triboelectric nanogenerator by charge space-accumulation effect. Nat. Commun..

[19] Shan C, He W, Wu H, Fu S, Tang Q (2022). A high-performance bidirectional direct current teng by triboelectrification of two dielectrics and local corona discharge. Adv. Energy Mater..

[20] Long L, Liu W, Wang Z, He W, Li G (2021). High performance floating self-excited sliding triboelectric nanogenerator for micro mechanical energy harvesting. Nat. Commun..

[21] Park D, Shin SH, Yoon IJ, Nah J (2018). Ferroelectric nanoparticle-embedded sponge structure triboelectric generators. Nanotechnology.

[22] Wang R, Mu L, Bao Y, Lin H, Ji T (2020). Holistically engineered polymer-polymer and polymer-ion interactions in biocompatible polyvinyl alcohol blends for high-performance triboelectric devices in self-powered wearable cardiovascular monitorings. Adv. Mater..

[23] Tan D, Zeng Q, Wang X, Yuan S, Luo Y (2022). Anti-overturning fully symmetrical triboelectric nanogenerator based on an elliptic cylindrical structure for all-weather blue energy harvesting. Nano-Micro Lett..

[24] Wang ZL, Wang AC (2019). On the origin of contact-electrification. Mater. Today.

[25] Qin K, Chen C, Pu XJ, Tang Q, He WC (2021). Magnetic array assisted triboelectric nanogenerator sensor for real-time gesture interaction. Nano-Micro Lett..

[26] Tang W, Chen BD, Wang ZL (2019). Recent progress in power generation from water/liquid droplet interaction with solid surfaces. Adv. Funct. Mater..

[27] Kim M, Park D, Alam MM, Lee S, Park P (2019). Remarkable output power density enhancement of triboelectric nanogenerators via polarized ferroelectric polymers and bulk MoS_2_ composites. ACS Nano.

[28] Wu H, Wang S, Wang Z, Zi Y (2021). Achieving ultrahigh instantaneous power density of 10 MW/m^2^ by leveraging the opposite-charge-enhanced transistor-like triboelectric nanogenerator (OCT-TENG). Nat. Commun..

[29] Wang K, Li J (2021). Electricity generation from the interaction of liquid-solid interface: a review. J. Mater. Chem. A.

[30] Li C, Liu X, Yang D, Liu Z (2022). Triboelectric nanogenerator based on a moving bubble in liquid for mechanical energy harvesting and water level monitoring. Nano Energy.

[31] Nie J, Ren Z, Xu L, Lin S, Zhan F (2020). Probing contact-electrification-induced electron and ion transfers at a liquid-solid interface. Adv. Mater..

[32] Erdemir A, Ramirez G, Eryilmaz OL, Narayanan B, Liao Y (2016). Carbon-based tribofilms from lubricating oils. Nature.

[33] Zhao J, Li Y, He Y, Luo J (2019). In situ green synthesis of the new sandwichlike nanostructure of Mn_3_O_4_/graphene as lubricant additives. ACS Appl. Mater. Interfaces.

[34] Zhao J, Huang Y, He Y, Shi Y (2021). Nanolubricant additives: a review. Friction.

[35] Wu G, Yang J, Shang J, Fang D (2020). A rotary fluid power converter for improving energy efficiency of hydraulic system with variable load. Energy.

[36] Chua MH, Cheng W, Goh SS, Kong J, Li B (2020). Face masks in the new covid-19 normal: materials, testing, and perspectives. Research.

[37] Wang J, He J, Ma L, Zhang Y, Shen L (2020). Multifunctional conductive cellulose fabric with flexibility, superamphiphobicity and flame-retardancy for all-weather wearable smart electronic textiles and high-temperature warning device. Chem. Eng. J..

[38] Zhao J, Wang D, Zhang F, Liu Y, Chen B (2021). Real-time and online lubricating oil condition monitoring enabled by triboelectric nanogenerator. ACS Nano.

[39] Nie J, Wang Z, Ren Z, Li S, Chen X (2019). Power generation from the interaction of a liquid droplet and a liquid membrane. Nat. Commun..

[40] Raadnui S, Kleesuwan S (2005). Low-cost condition monitoring sensor for used oil analysis. Wear.

[41] Du L, Zhe J, Carletta J, Veillette R, Choy F (2010). Real-time monitoring of wear debris in lubrication oil using a microfluidic inductive coulter counting device. Microfluid Nanofluidics.

[42] Droubi MG, Reuben RL, Steel JI (2018). Flow noise identification using acoustic emission (AE) energy decomposition for sand monitoring in flow pipeline. Appl. Acoust.

[43] Baghelani M, Hosseini N, Daneshmand M (2021). Non-contact real-time water and brine concentration monitoring in crude oil based on multi-variable analysis of microwave resonators. Measurement.

[44] Liu P, Liu S, Yu X, Zhang Y (2021). Silane-triggered fabrication of stable waterborne superamphiphobic coatings. Chem. Eng. J..

[45] Li Q, Guo Z (2019). A highly fluorinated SiO_2_ particle assembled, durable superhydrophobic and superoleophobic coating for both hard and soft materials. Nanoscale.

[46] Pan Y, Huang S, Li F, Zhao X, Wang W (2018). Coexistence of superhydrophilicity and superoleophobicity: theory, experiments and applications in oil/water separation. J. Mater. Chem. A.

[47] Xie X, Chen X, Zhao C, Liu Y, Sun X (2021). Intermediate layer for enhanced triboelectric nanogenerator. Nano Energy.

[48] Hughes C, Yeh LH, Qian S (2013). Field effect modulation of surface charge property and electroosmotic flow in a nanochannel: stern layer effect. J. Phys. Chem. C.

[49] Wang ZL, Lin L, Chen J, Niu S, Zi Y (2016). Triboelectric Nanogenerators..

[50] Jiang P, Zhang L, Guo H, Chen C, Wu C (2019). Signal output of triboelectric nanogenerator at oil-water-solid multiphase interfaces and its application for dual-signal chemical sensing. Adv. Mater..

[51] Clermont PDS, Paillat T, Peres G, Duval Y, Touchard G (2017). Quantification of flow electrification in the context of a tank being filled with a dielectric liquid. IEEE Trans. Ind. Appl..

[52] Baytekin HT, Baytekin B, Soh S, Grzybowski BA (2011). Is water necessary for contact electrification?. Angew. Chem. Int. Ed..

[53] Ishikawa T, Yasuda K, Igarashi T, Yanabu S, Ueta G (2009). Effect of temperature on the streaming electrification characteristics of silicone oil. IEEE. Trans. Dielectr. Electr. Insul..

[54] Chu Z, Feng Y, Seeger S (2015). Oil/water separation with selective superantiwetting/superwetting surface materials. Angew. Chem. Int. Ed..

[55] Yao X, Song Y, Jiang L (2011). Applications of bio-inspired special wettable surfaces. Adv. Mater..

[56] Myshkin NK, Markova LV (2018). On-line Condition Monitoring in Industrial Lubrication and Tribology..

[57] Cantley RE (1977). The effect of water in lubricating oil on bearing fatigue life. ASLE Transact..

[58] Smiechowski MF, Lvovich VF (2002). Electrochemical monitoring of water-surfactant interactions in industrial lubricants. J. Electroanal. Chem..

[59] Urban A, Zhe J (2022). A microsensor array for diesel engine lubricant monitoring using deep learning with stochastic global optimization. Sens. Actuator A Phys..

