# Supplementary material for: Self-Powered, Long-Durable, and Highly Selective Oil–Solid Triboelectric Nanogenerator for Energy Harvesting and Intelligent Monitoring
Source: Nanomicro Lett. 2022 Aug 5;14:160. doi: 10.1007/s40820-022-00903-8 (PMC9356124; doi:10.1007/s40820-022-00903-8)
Supplement: Supplementary file 7 — Supplementary file7 (PDF 1858 KB) [file 40820_2022_903_MOESM7_ESM.pdf]

Supporting Information for

Self-Powered, Long Durable, and Highly Selective Oil-Solid Triboelectric Nanogenerator for Energy Harvesting and Intelligent Monitoring

Jun Zhao[1], Di Wang[1], Fan Zhang[2], Jinshan Pan[3], Per Claesson[3], Roland Larsson[1], Yijun Shi[1, *]

[1]Division of Machine Elements, Department of Engineering Sciences and Mathematics, Luleå University of Technology, Luleå, SE-971 87, Sweden

[2]Department of Engineering and Design, School of Engineering and Informatics, University of Sussex, Brighton, BN1 9RH, United Kingdom

[3]Division of Surface and Corrosion Science, Department of Chemistry, KTH Royal Institute of Technology, Stockholm, SE-100 44, Sweden

*Corresponding author. E-mail: yijun.shi@ltu.se (Yijun Shi)

Supplementary Figures and Tables

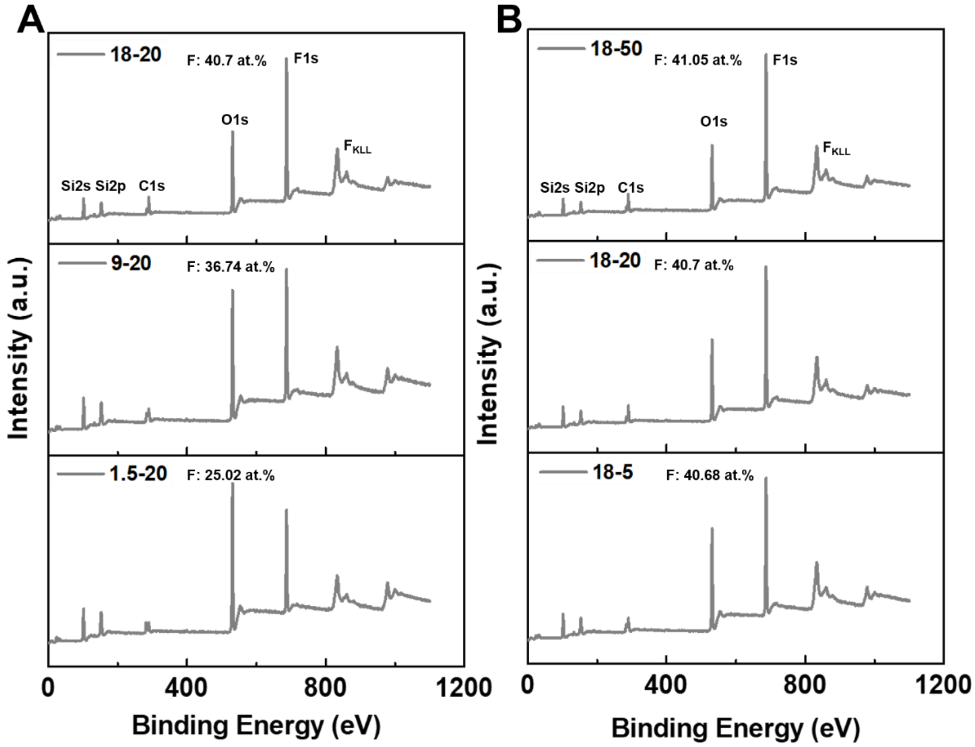

Fig. S1 XPS wide-scan spectra of FO-TENGs with different Fc fraction (**A**) and with different Fs faction (**B**)

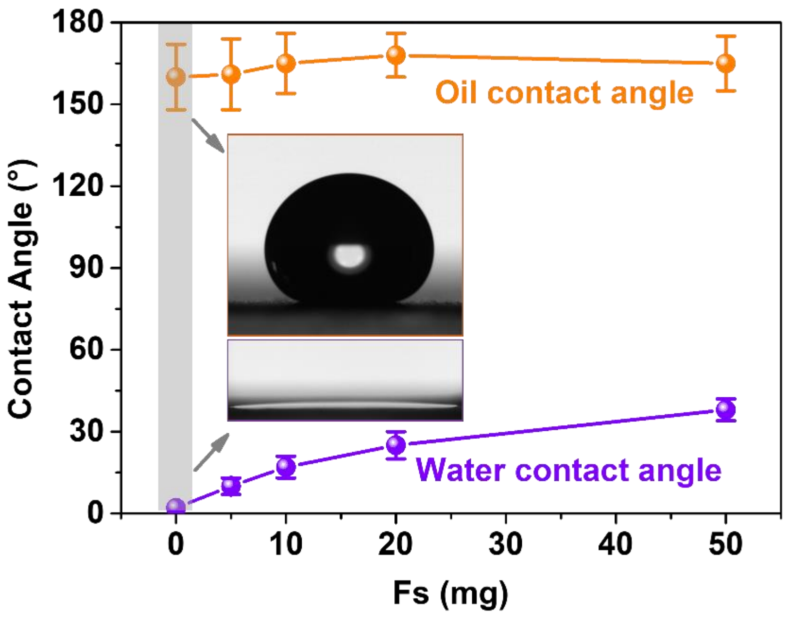

Fig. S2 Contact angles (oil and water) on FO-TENGs as a function of Fs mass (Fc: 18 g). Inset: paraffin oil and deionized water contact angle images

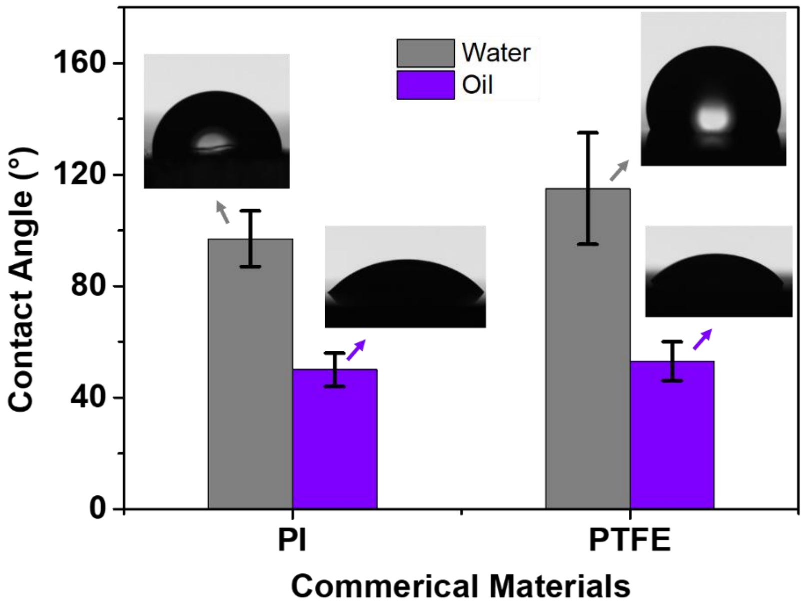

Fig. S3 Contact angles (oil and water) on commercial pure PI and PTFE, inset: paraffin oil and deionized water contact angle images

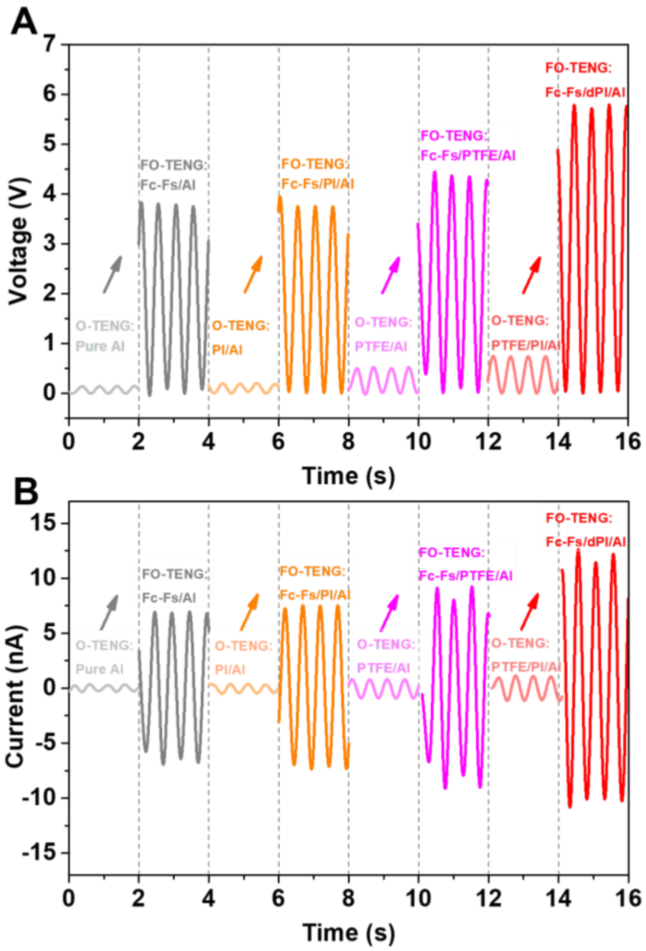

Fig. S4 The open-circuit voltage (**A**) and the short-circuit (**B**) for different O-TENGs. The dPI in the Fc-Fs/dPI/Al is from a double PI tape

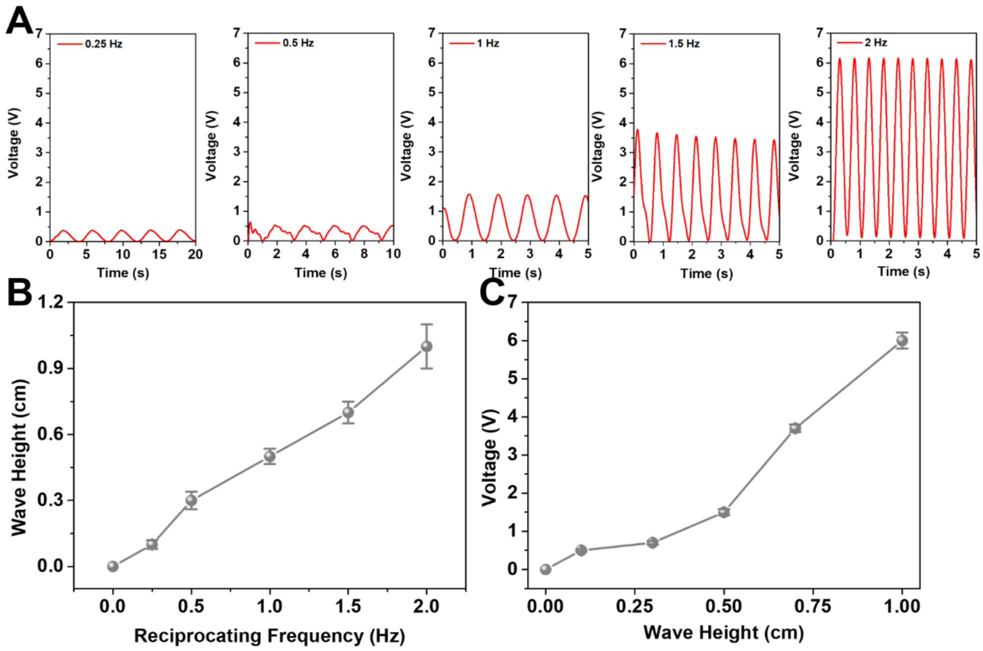

Fig. S5 The open-circuit voltages of the FO-TENG (18-20) (**A**) at different reciprocating frequencies. The oil wave height as a function of the tank reciprocating frequency (**B**). The open-circuit voltage output of the FO-TENG at different oil wave heights (**C**)

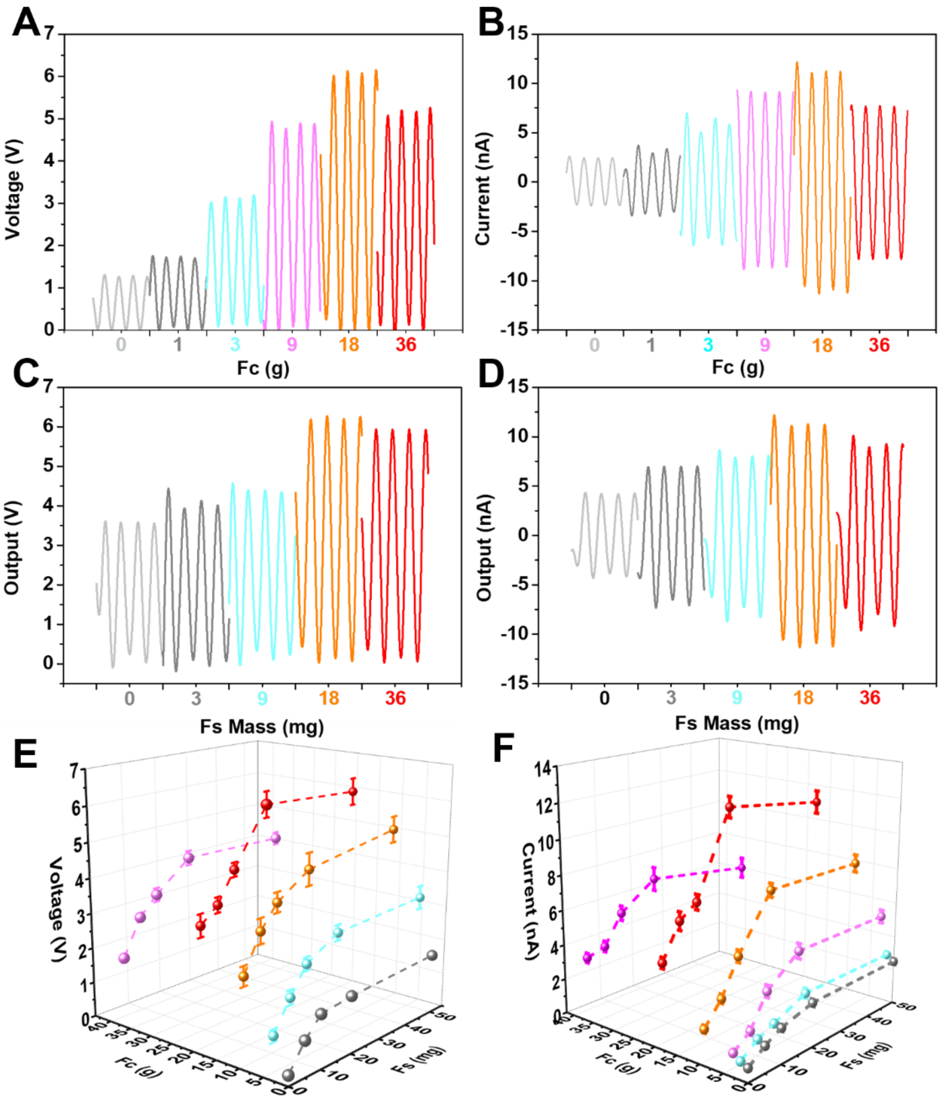

Fig. S6 The open-circuit voltage (**A**) and short-circuit current (**B**) of the FO-TENG at different Fc mass. The open-circuit voltage (**C**) and short-circuit current (**D**) of the FO-TENG at different Fs mass. The open-circuit voltage (**E**) and short-circuit current (**F**) of the FO-TENG at different Fc and Fs mass

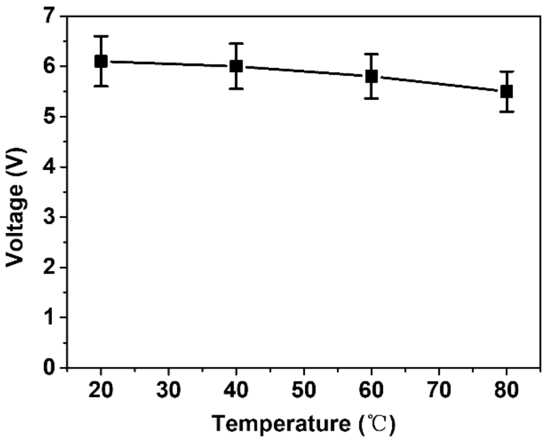

Fig. S7 Output voltage of the FO-TENG (18-20) contacting paraffin oil as a function of oil temperature

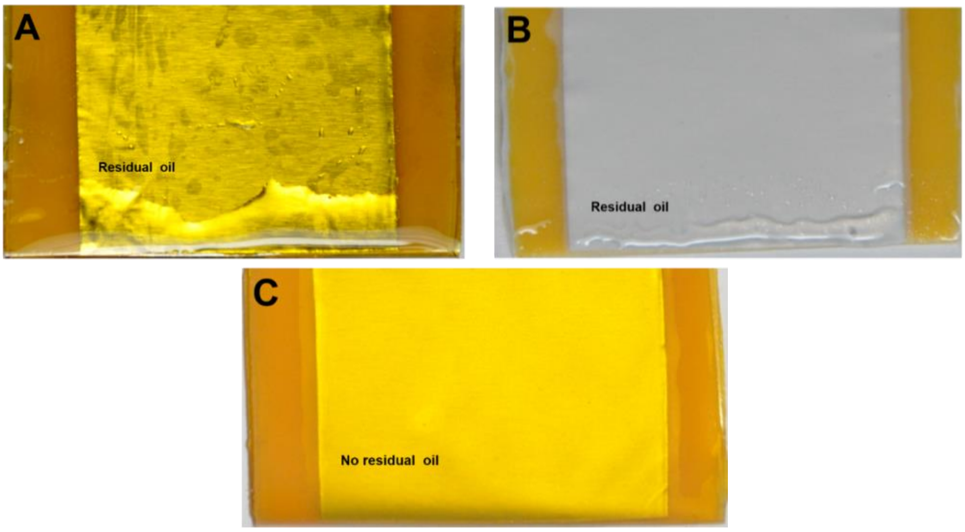

Fig. S8 Optical microscopy images of the PI/Al (**A**), PTFE/Al (**B**) O-TENGs and FO-TENG (18-20) (**C**) after 30 000 cycles

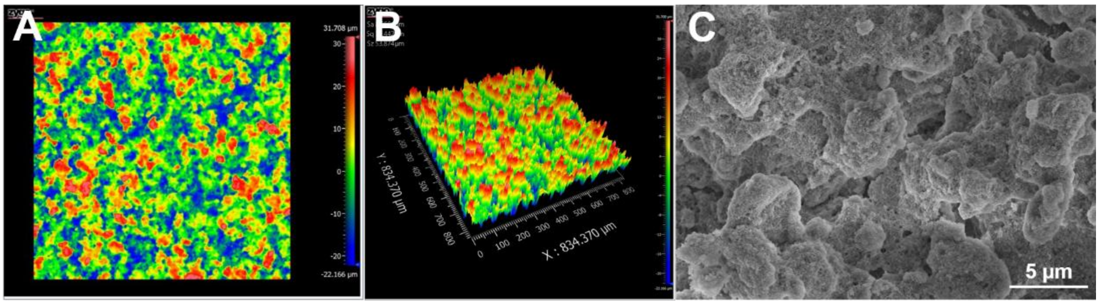

Fig. S9 Two-dimensional (**A**), three-dimensional (**B**) and SEM (**C**) morphologies of the FO-TENG surface (18-20) after 30,000 cycles

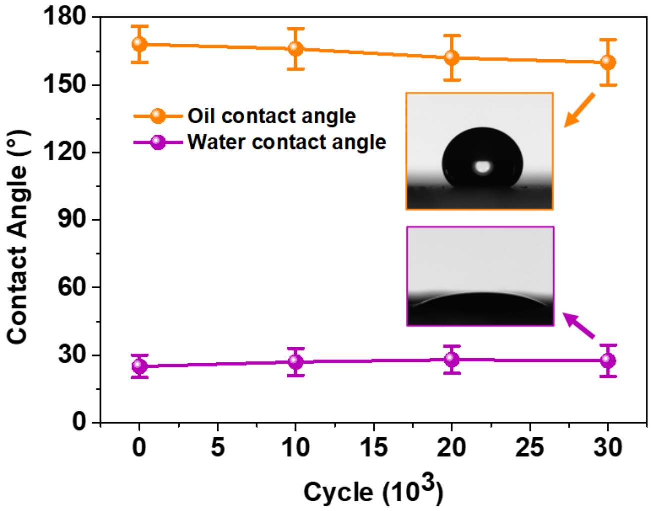

Fig. S10 Contact angles (oil and water) on the FO-TENG (18-20) as a function of working cycle. Inset: paraffin oil and deionized water contact angle images

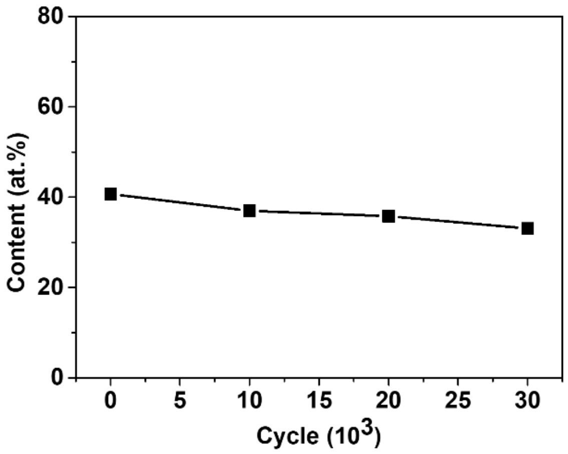

Fig. S11 F content of the FO-TENG (18-20) surface by XPS analysis as a function of working cycle

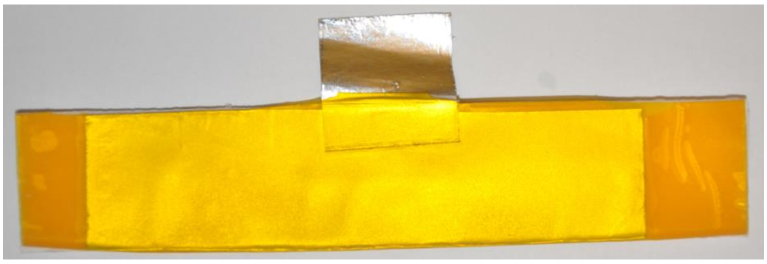

Fig. S12 The optical image of the FO-TENG (18-20) with a larger size (8 cm × 2 cm)

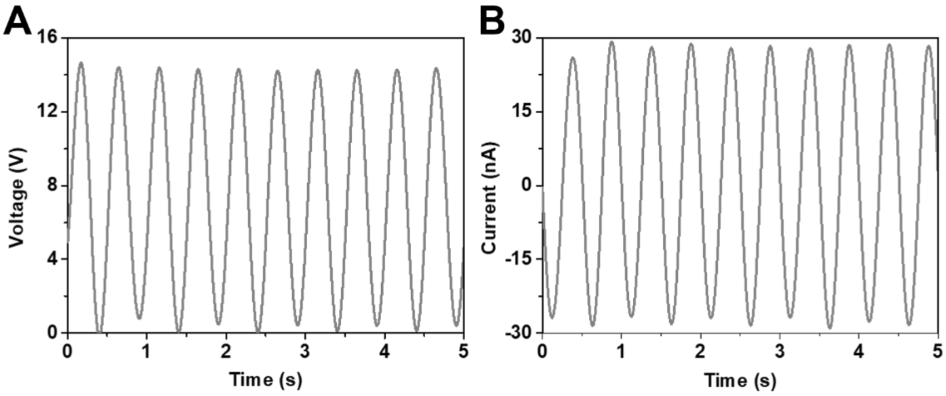

Fig. S13 The open-circuit voltage (**A**) and the short-circuit (**B**) of the FO-TENG (Fig. S12) based on single electrode mode

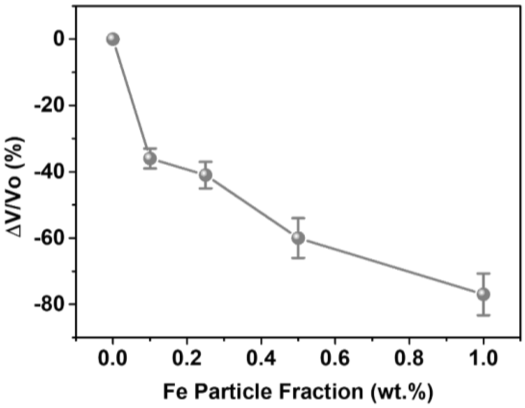

Fig. S14 The voltage output of the FO-TENG (18-20) contacting Fe particle-ladened oil

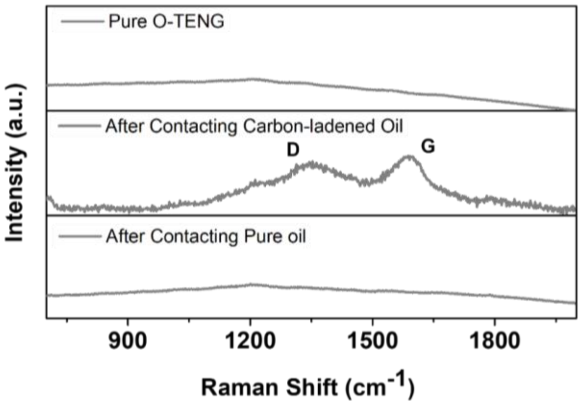

Fig. S15 The Raman Spectra of the FO-TENG (18-20) contacting contaminant-laden oil and pure oil. The D (1348.3cm^{-1}) and G (1588 cm^{-1}) peaks are characteristic peaks of graphite from carbon black

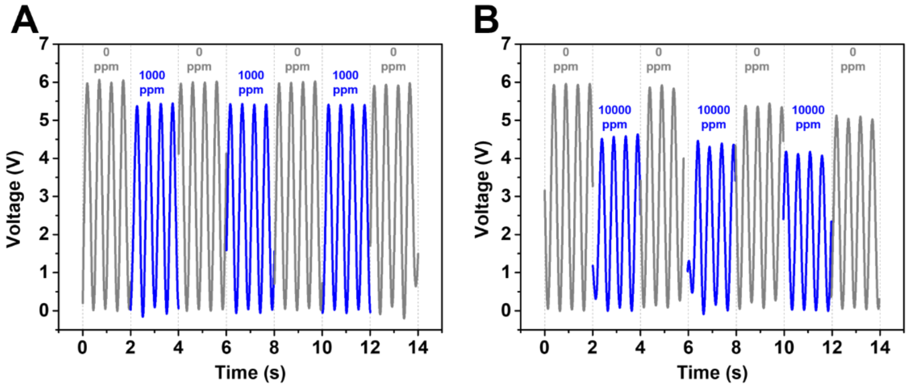

Fig. S16 Reversible self-cleanability of the FO-TENG (18-20) contacting water contaminant oil

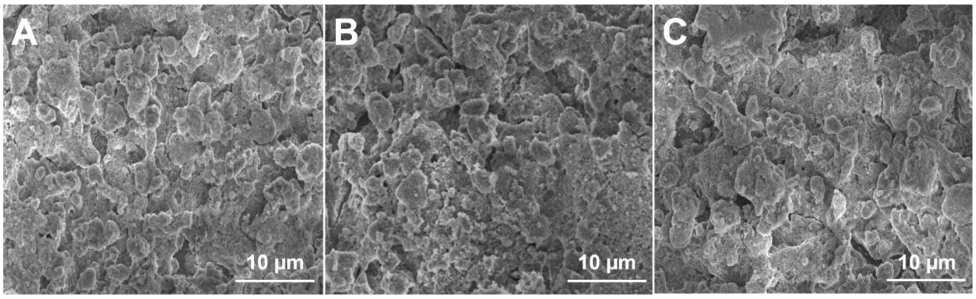

Fig. S17 SEM morphologies of the original FO-TENG (18-20) (**A**) and those contacting the water contaminant oils (1000 ppm) (**B**) and (10000 ppm) (**C**) after three cycles

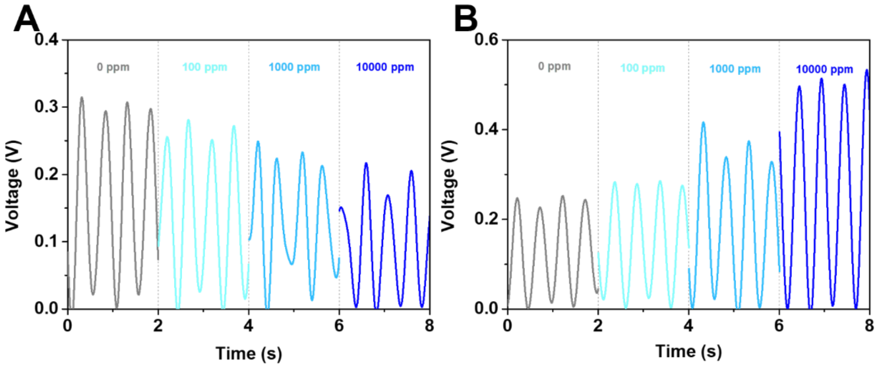

Fig. S18 The voltage outputs of FO-TENG (18-20) (**A**) and FO-TENG (1.5-20) (**B**) contacting a commercial engine lubricating oil with water contaminant

Table S1 Content of materials in the SiO_2 suspension, where n represents the content of Fs

Name	Water (g)	SiO_2 (g)	Fc (g)
0-n			0
1.5-*n*			1.5
3-*n*	30	1	3
9-*n*			9
18-*n*			18
36-*n*			36

Table S2 Content of materials in the SiO_2 suspension, where m represents the content of Fc

Name	Water (g)	SiO_2 (g)	Fs (mg)
m-0			0
m-5			5
m-10	30	1	10
m-20			20
m-50			50

Table S3 Element contents (atom%) of O-TENGs surfaces calculated from XPS spectra

TENGs	C	O	F	Si	N
FO-TENG (1.5-20)	21.59	36.74	25.02	16.1	0.55
FO-TENG (9-20)	22.45	28.13	36.76	11.7	0.96
FO-TENG (18-20)	24.66	25.43	40.7	8.5	0.71
FO-TENG (18-5)	23.89	27.5	40.68	7.1	0.83
FO-TENG (18-50)	25.77	23.7	41.05	8.8	0.68
O-TENG (PI/Al)	74.09	13.89	9.57	0.2	2.25
O-TENG (PTFE/Al)	53.99	9.01	36.32	0.1	0.58
